# Methodological concerns restrict generalizations about children’s overimitation

**DOI:** 10.3389/fpsyg.2026.1909142

**Published:** 2026-10-15

**Authors:** Ashley B. Marin, Rebekah A. Richert, Frankie T. K. Fong

**Affiliations:** 1 Department of Psychological and Brain Sciences, University of California, Riverside, Riverside, CA, United States; 2 School of Psychological Sciences, Victoria University of Wellington, Wellington, New Zealand; 3 Department of Comparative Cultural Psychology, Max-Planck-Institut fur evolutionare Anthropologie, Leipzig, Germany

**Keywords:** children, cultural transmission, cumulative culture, imitation, overimitation, social learning

## Abstract

Overimitation has been studied and theorized as a key social learning mechanism that drives cultural transmission. Despite notable theoretical advancements, methodological inconsistencies and ecological gaps have made it difficult to draw broad conclusions about the nature and function of overimitation. These differences create confounding explanations for both the factors that influence overimitation, as well as the developmental and cultural functions of overimitation, leaving gaps in what either individual studies or the collective body of work can reliably say about the causes and purposes of overimitation. That is, certain effects may be partly, and unintentionally, shaped by specific methodological or procedural details. In this article, we discuss how variations in design components from existing studies create tensions for explanations and conclusions. We focus on the experimental tasks themselves, the behavioral coding schemes used to evaluate overimitation, and how experiments are set up. We conclude by proposing approaches that place both the research question and the cultural context at the forefront, enabling scholars to more accurately study and/or compare children’s imitative behaviors across cultures and contexts.

## Introduction

Over the past two decades, numerous theoretical advances emerged from empirical research studying overimitation^1^ – the near-identical reproduction of another individual’s actions from the beginning to the end of a modeled behavioral sequence (Hoehl et al., 2019). Scholars have examined the expression of overimitation across and between age groups and within caregiver–child dyadic contexts, while also testing its universality across different cultural ecologies and its contextual sensitivity (e.g., Décaillet et al., 2024; Mackie et al., 2025; Nielsen and Tomaselli, 2010; Papa et al., 2021). The diversity of investigative approaches have produced a range of explanations for why individuals reproduce causally-redundant behaviors with high fidelity, emphasizing specific social, normative, pedagogical, cognitive, and instrumental mechanisms (Gergely and Csibra, 2020; Keupp et al., 2013; Nielsen, 2018; Schleihauf and Hoehl, 2020). By extension, these accounts and lines of inquiry point toward evolutionary functions of overimitation, including its role in supporting the transmission and acquisition of technical skills and conventional practices, maintaining child-caregiver relations, fostering shared goals, and supporting group competency (Lakin et al., 2003; Legare and Nielsen, 2015; Over, 2020).

Despite this cumulative progress, the field remains methodologically heterogeneous, with substantial variation in experimental paradigms, task structures, and coding approaches. This variability remains an active constraint on integrating conclusions about stable versus context-dependent mechanisms and the functions of overimitation. In particular, subtle differences in methodological details may inadvertently guide observed behavioral patterns, leaving some alternative explanations underexamined. For example, Fong and Haun (2025) discussed how pedagogical cues within an experimental session, as well as during a lab visit in general, may influence overimitation. Relatedly, opportunities remain to better align experimental paradigms with features of the social ecologies in which children holistically learn from others. In this paper, we concentrate on three core dimensions of experimental design: (1) task structure, (2) coding schemes, and (3) experimental setup.

Our aim is to catalyze more attention to the quiet ways experimental design affects participant performance, an often-unintentional pattern famously highlighted by Rosenthal and Jacobson (1968) in *Pygmalion in the Classroom*. We presume most researchers have considered these kinds of demand characteristics (Orne, 1962) at least once while designing their research protocols; and Hoehl et al. (2019) formally addressed the issue identifying sources of experimental variation (i.e., hidden factors) that may influence participant behavior, misguide interpretations of results, and constrain comparability across studies. By integrating methodological recommendations with a broader consideration of cultural context and ecological validity, we aim to support a more unified framework for studying overimitation and a more complete understanding of the role of overimitation in development, cultural transmission, and human cognitive evolution.

### Task structure and ecological correspondence

The vast majority of existing overimitation studies have used a puzzlebox, wherein participants observe a sequence of causally-irrelevant actions (e.g., tapping the top 3 times) followed by a causally-relevant action (e.g., opening a door flap) to open a box with a tool in order to achieve the end goal of retrieving an object (e.g., Horner and Whiten, 2005; Nielsen and Tomaselli, 2010). Alternative approaches integrate the use of materials or tasks that resemble everyday activities, such as activating light switches, turning clocks, and manipulating household items like jars, hair ties, and spoons (e.g., Keupp et al., 2013; Pauen and Bach, 2025; Stengelin et al., 2022). Researchers have also situated tasks within socially-meaningful contexts by incorporating peer-game interactions, parent demonstrators, and natural settings (e.g., home, public spaces) (Bach and Pauen, 2026; Mackie et al., 2025; Whiten et al., 2016). Our review of this work has highlighted two key areas across experimental tasks where ecological validity can be meaningfully considered.

#### Non-observable outcomes in real world experience

The most common experimental designs involve tasks with a visually evident end goal, mirroring the nature of a particular kind of action sequence in which some action(s) lead to clear, observable consequences. This emphasis is not necessarily misplaced, as many activities children engage in involve goals whose successful completion can be readily perceived. Yucatec Mayan children, for example, often engage in activities such as cleaning, assisting with construction projects, and preparing poultry for consumption, where successful completion is indicated by observable outcomes (Alcalá and Cervera, 2022).

In contrast, few, if any, paradigms focus on action sequences that do not aim to result in either a tangible end-product (e.g., making a necklace) or obtaining a reward (e.g., the object inside the puzzle box) – the kinds of action sequences that permeate cultural practices. Cultural and religious rituals incorporate sequences of symbolic actions that rarely produce immediately perceptible causal effects (Fiese et al., 2002; Zhao et al., 2025). Nauhua children, for example, participate in Day of the Dead celebrations by helping prepare bread offerings for ancestors (Bowen, 2024). While these action sequences resemble the everyday functional tasks of cooking, they are situated within a ritualistic context in which the outcome serves a spiritual purpose that is not physically observable, such as welcoming the spirits of ancestors. For studies of overimitation to more fully speak to mechanisms of cultural evolution and cultural learning, advancements are needed in the design of tasks that have non-observable outcomes.

#### Those modeling the action sequence

Researchers have typically used unfamiliar adult experimenters as task demonstrators. Yet, a growing body of studies have started to examine children’s learning from more ecologically-valid social partners by including parents (Mackie et a., 2025; Pauen and Bach, 2025), puppets (Dragon and Poulin-Dubois, 2023; Stengelin et al., 2022), robots (Hu and Li, 2025; Schleihauf et al., 2021; Sommer et al., 2020), and familiar teachers (McGuigan, 2013; Wang and Wang, 2023) as demonstrators. Findings are mixed, with some studies reporting greater overimitation of familiar models and others showing a preference for unfamiliar or non-human demonstrators.

Collectively, these studies reaffirm that children’s overimitation is not only context-dependent but also is influenced by their relationship to and assumptions about the model, suggesting that model-based biases function as evolutionarily-adaptive shortcuts for efficiently acquiring useful information (Fong et al., 2023; Wood et al., 2013). These findings highlight the critical need for more careful delineation of the effects of who children observe perform the target action sequence. This limitation may be particularly relevant in contexts where learning is distributed across multiple community members, who contribute to children’s acquisition of culturally valued skills through multiple interactions. For instance, in Mexican and Central American immigrant families in the United States, extended caregiving systems are common, with older siblings and grandparents supporting children’s academic and cultural competency through daily homework assistance and scaffolding of traditions and customs (Fuller-Thomson and Minkler, 2007; Orellana, 2001). Another critical consideration in the current historical context is the role of artificially intelligent, media-based, and robotic agents in social learning, as children’s environments are increasingly including novel anthropomorphic agents, with families reporting the use of AI-based tools for homework support (Kirkorian et al., 2022; Wang et al., 2026).

Another factor is the extent to which participants perceive themselves as similar to, or different from, the researcher modeling the behavior. Theorists have speculated perceived similarity signals others’ behaviours are especially relevant within a shared group identity (Smaldino and Velilla, 2025; Wood et al., 2013). Researchers have considered “experimenter effects” through gender counterbalancing and/or analyses of gender effects in overimitation experiments (Décaillet et al., 2024; Pauen and Bach, 2025; Zhao et al., 2025). Studies have also examined identity cues such as race, shared-language, and minimal group markers (e.g., shirt color) (Altınok et al., 2022; Krieger et al., 2020; Schleihauf et al., 2019).

Additionally, the level of rapport established between the demonstrator and participant prior to starting the study may contribute to perceived similarity. Initial shared interactions may allow children to perceive similarity beyond cues explicitly considered by the research team (Schleihauf et al., 2019). Studies vary considerably in the extent of pre-task interactions, and these are typically carried out by the same experimenter who performs the demonstration. Of note, these kinds of interactions are rarely described in methods sections. Consequently, there are many possible dimensions along which similarity may be formed, and it is often unclear which aspects are most salient to participants. Studies should carefully consider the nature of who is modeling the action sequence, even when the effect of the model is not the central research concern.

### Values and assumptions in coding schemes

A typical overimitation coding scheme focuses on whether an action is being replicated or not, irrespective of the specific method used (Hoehl et al., 2019). Imagine a demonstrator using three fingers to hold the middle of a stick and tap the side of the box three times. A child would likely be coded for overimitation if they use their whole hand to grasp a different part of the stick, as long as they tap. Similarly, participants who reproduce the action once, three times, or five times would receive identical scores under binary coding schemes. Many coding approaches also do not consider action order. Consequently, participants who produce the same set of actions but in substantially different sequences may receive equivalent scores. These examples may be categorised as a form of “process emulation,” in which the process is being replicated but not via the exact same means (Heyes, 2021). Similarly, a goal can be achieved with a different visible outcome. For example, considering a task that involves placing a kettle on a table, one may be considered as successfully achieving this goal as long as it is being “placed” but in certain cultures/contexts, it may be crucial that the kettle is placed at the exact same spot facing a specific direction. Indeed, coding decisions may also reflect implicit assumptions about what counts as successful overimitation, including whether imitation is defined in terms of reproducing actions, outcomes, or the broader process, and whether approximate attempts or precise replication is valued.

#### Precision, repetition, and sequence

Many cultural practices value precision (i.e., how closely each individual action matches the demonstrated manner of execution), repetition (i.e., the number of times an action should be performed), and sequence (i.e., the order in which actions should occur). For example, in Japan, the degree of bows depends on the context or the person involved, and the exact spatial organization of eating utensils is critical in many cultural settings (Okado et al., 2018; Zampollo et al., 2012). Similarly, prayer practices can involve structured repetition and ordered sequences of actions, including being performed a specified number of times per day and following procedural sequences in which preparatory actions, such as spiritual purification practices, precede a prayer (Mazumdar and Mazumdar, 2004). These three features represent cultural nuances that coding techniques focused on the mere presence or absence of actions cannot readily accommodate.

Methodologically, capturing and reliably coding precision, repetition, and/or sequence often require more fine-grained observational and coding protocols than simply scoring whether target actions are reproduced. Although ensuring that tasks are developmentally appropriate for the age group being studied is essential, this focus does not preclude further exploration of more nuanced aspects of action reproduction within tasks already considered suitable for children of a given age. Whether these dimensions reflect limitations in children’s competencies or differences in how children attend to and reproduce observed actions remains largely unexplored. However, understanding when and why children incorporate these aspects of overimitation into their behavioral sequences is critical for connecting experimental work to the lived contexts in which overimitation functions for cultural learning.

### Experimental setup design

Across experimental designs, some decisions and manipulations appear to produce relatively replicable patterns in overimitation. For instance, there is considerable evidence that exploration or familiarity with the apparatus does not eliminate overimitation (e.g., Nielsen and Tomaselli, 2010; Marsh et al., 2019; Rizvanović et al., 2025). Besides studies designed specifically to test the effect of verbal cues (Clegg and Legare, 2016; Herrmann et al., 2013), overimitation studies tend to include minimal instructions for children (e.g., “watch this”). Despite the consistency of major aspects of an experiment, we pinpoint three aspects of the ways a task is set up for children in which substantial variation and ambiguity remain.

#### Participants’ sporadic linguistic input

The presence of questions from child participants in overimitation experiments is almost inevitable among children given their natural tendency to ask more questions in contexts where need for information is high, including when physical causality is unclear (Callanan and Oakes, 1992; Tippenhauer et al., 2026). A common practice in overimitation paradigms is to provide standardized prompts in response to participant questions, rather than engaging in free-form response during the task. One commonly used prompt is “do it however you want” or similar variations (Allen et al., 2021; Pauen and Bach, 2025; Wilks et al., 2019). Other approaches include prompts such as “go ahead and try it out,” or “go on, just give it a try” (Keupp et al., 2013; Rizvanović et al., 2025).

While these prompts validate that questions occur in these experiments, they also bring attention to the likelihood that other forms of participant input are taking place outside of questions. Mackie et al. (2025) documented cases of attempts to “correct” the model during the experiment. How teams respond to other types of participant input are comparatively underreported and may not be consistently addressed across studies. Different kinds of participant input (e.g., inquiring about material ownership versus why things are done a certain way) may produce variation in experimenter responses (e.g., ignoring the question versus providing a prompted or free response), unintentionally creating feedback loops (Warlaumont et al., 2014), in which children’s input influences experimenter responses that in turn shape participant behavior. For a deeper understanding of the role and agency of children in cultural learning, it is critical for researchers to share the kinds of questions, responses, and reactions children articulate within the experimental context.

#### Reward effects

Reward types – consumable treats, token-based reinforcers (e.g., stickers), physical items (e.g., toys), etc. – are situated within many task structures, with engagement often involving manipulation of objects to physically obtain a reward. Similarly, in assemblage-oriented tasks in which individuals construct an object, the presence of desirable materials and the absence of explicit guidance regarding ownership of the final product may signal an opportunity of reward acquisition. Two considerations arise. First, differences in the subjective value of rewards and materials may affect participants’ motivation, attention, and interpretation of task goals, influencing their behavior. Second, ambiguity regarding rewards – not specifying whether participants may keep the materials or whether acquisition is contingent upon participation or task success – may support extrinsic motivation. Research on token economies within educational settings underscores how extrinsic rewards can effectively influence motivation and behavioral outcomes in learning contexts (Kim et al., 2022). Subsequently, overimitation scores may also reflect underlying mechanisms of extrinsic motivation rather than purely social or affiliative influences, or some combination of these factors.

Importantly, the presence of rewards within overimitation tasks do reflect ecologically valid aspects of real-world learning environments. Caregivers often structure opportunities for incentives to encourage children to engage in everyday tasks. Among European American mothers with high levels of school experience, a strategy for encouraging toddlers’ participation in household chores is the use of rewards contingent on chore completion (Coppens and Rogoff, 2022). Rewards can also differ in their temporal placement relative to task engagement. Children from the Hadza hunter-gatherers of Tanzania commonly consume gathered foods after foraging activities, linking reward immediately to the task itself (Crittenden et al., 2013). Given these kinds of variations in the presence and role of rewards in children’s cultural learning, studies of overimitation should consider the timing, nature, and transparency of rewards to children.

## Discussion

Scholars studying overimitation have made important theoretical advances that reveal insights into our understanding of the evolutionary mechanisms underlying the phenomenon. However, persistent variability in task structure, coding practices, and experimental design hinder clear interpretation and integration of findings into a coherent understanding of overimitation and its developmental functions. Methodological differences place constraints on determining whether observed results reflect developmental or contextual processes or instead arise from variation in how the behavior is elicited, operationalized, and measured across studies. By extension, methodological decisions regarding task design may provide opportunities to better capture features of the social contexts in which children compose their behavior repertoires.

Important advances have begun to address some of these methodological concerns. Recent work has diversified task paradigms, incorporating unobservable end-goal features, comparing different end-goal contexts, and varying the type of demonstrator, including familiar and unfamiliar human models as well as non-human agents (e.g., Nielsen, 2018; Pauen and Bach, 2025; Schleihauf et al., 2021; Wang and Wang, 2023). Coding practices have evolved toward approaches that extend beyond binary classification of presence or absence of certain behaviors, including proportion-based scoring that distinguishes between approximate and exact reproductions of demonstrated frequencies (e.g., McGuigan et al., 2011). Scholars have further developed a range of experimental approaches to better account for reward-related effects, such as more explicit task controls (e.g., incorporating a ‘re-storage’ component at the end of the imitation task) and systematic assessment of reward exposure factors (e.g., measuring preferences) (Pauen and Bach, 2025; Zhao et al., 2024).

### Recommendations for researchers

We propose an “IMITATE” reflective guide (Figure 1) and accompanying experimental checklist (Figure 2) to support the planning of overimitation studies. Both aim to increase methodological coherence across the literature by encouraging transparency in methodological decisions and procedures, while also helping researchers consider the fit between experimental designs and participants’ ecological contexts. The checklist also provides practical considerations to support researchers in making and clearly documenting these methodological decisions.

**Figure 1:**
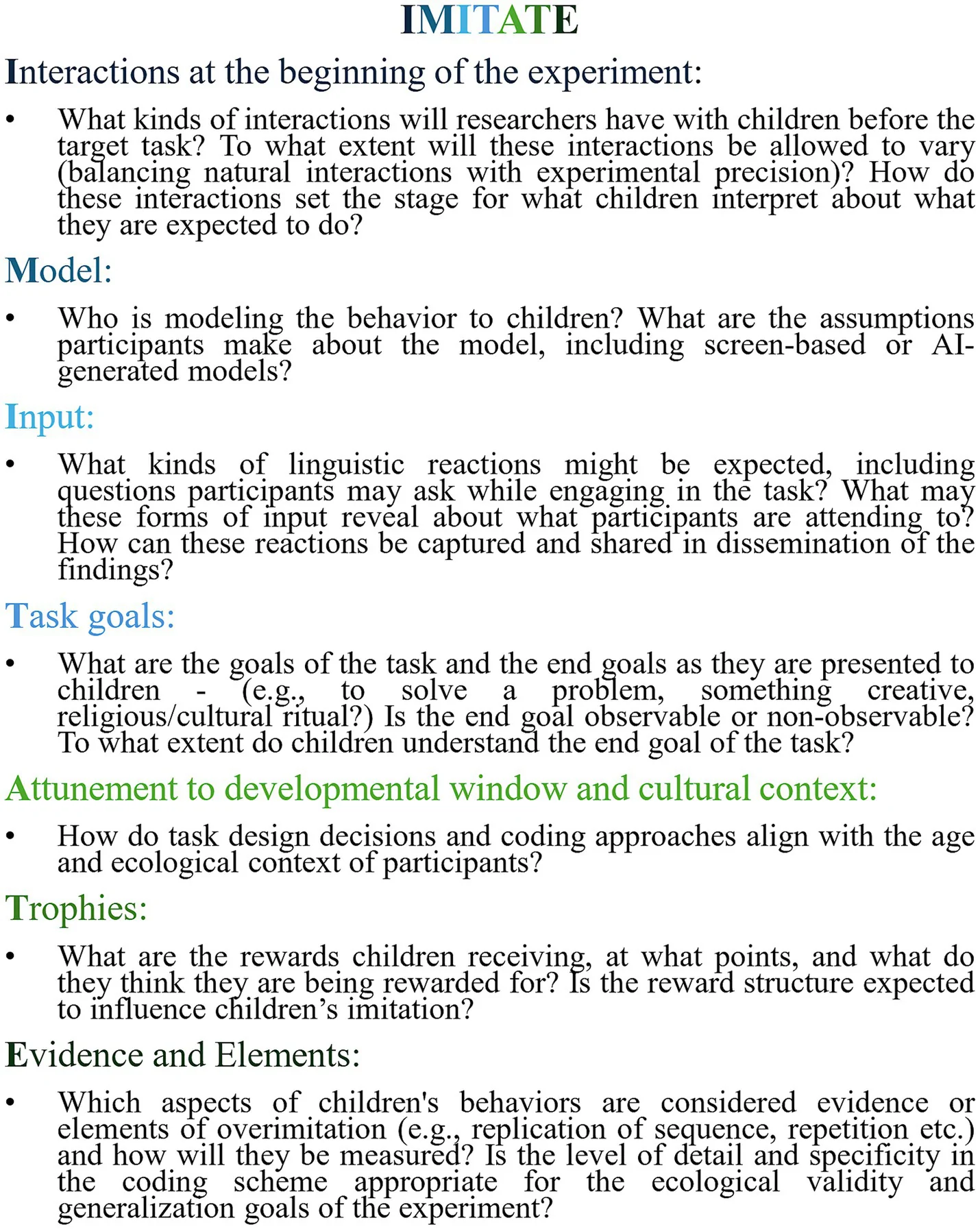
IMITATE reflective guide for planning overimitation studies.

**Figure 2:**
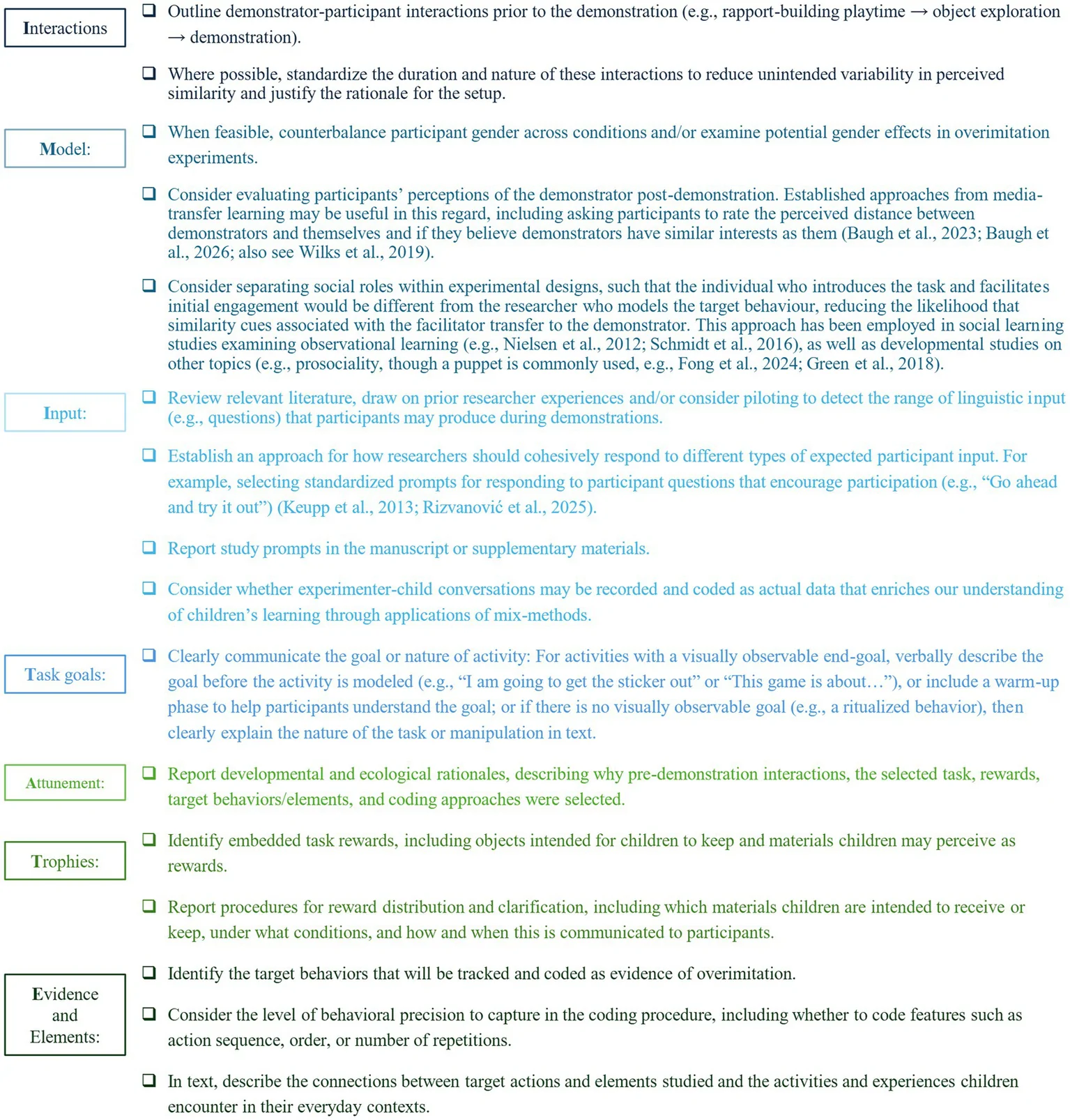
IMITATE Experimental checklist.

## Conclusion

Despite advances in rigorous experimental research examining children’s overimitation, the real-life dynamics and varying psychological processes that occur when a child is engaging in overimitation remain understudied. Understandably, researchers often place greater emphasis on key manipulations or variables that are directly related to the research question(s), where other components obtain less attention. As described above, a more granular approach is needed to enhance understanding of how overimitation functions and varies across children’s daily living contexts (e.g., rituals in religious and non-religious settings, home vs. school ecologies) and how overimitation serves as an efficient strategy for rapid acquisition of instrumental knowledge (e.g., language, problem-solving methods). The growing presence of socially interactive technologies in children’s everyday environments affirms the need to consider how these emerging agents may influence children’s social learning. Ongoing methodological refinements, with increasing coherence and rational clarity, promise to further unveil the psychological mechanisms that underpin cultural evolution across diverse childhood ecologies.

## Data Availability

The original contributions presented in the study are included in the article/supplementary material, further inquiries can be directed to the corresponding author.

## Footnotes

1 This paper focuses on overimitation, which is typically observed and studied in 3–4 year olds and older children. This form of social learning is different from (faithful) imitation in toddlers or infants. For more discussion see Fong et al. (2023).
